# Cerebrospinal fluid and serum biomarkers in idiopathic intracranial hypertension: A systematic review

**DOI:** 10.1111/head.15023

**Published:** 2025-08-08

**Authors:** Marina Romozzi, Fabio Zeoli, Renata Martinelli, Federico Tosto, Antonio Funcis, Giuseppe Garignano, Sabrina Chiloiro, Lucia Di Nardo, Catello Vollono, Alessandro Olivi, Paolo Calabresi, Francesco Signorelli

**Affiliations:** ^1^ Department of Neuroscience Università Cattolica del Sacro Cuore Rome Italy; ^2^ Neurology Unit, Dipartimento di Neuroscienze, Organi di Senso e Torace Fondazione Policlinico Universitario Agostino Gemelli IRCCS Rome Italy; ^3^ Department of Neurosurgery, Fondazione Policlinico Universitario Agostino Gemelli IRCCS Università Cattolica del Sacro Cuore Rome Italy; ^4^ Department of Neuroscience “Giovanni Paolo II” Hospital Catanzaro Italy; ^5^ Radiology and Neuroradiology Unit, Dipartimento di Diagnostica per Immagini, Radioterapia Oncologica ed Ematologia Fondazione Policlinico Universitario Agostino Gemelli IRCCS Rome Italy; ^6^ Dipartimento Di Medicina Traslazionale Università Cattolica del Sacro Cuore Rome Italy; ^7^ Dipartimento Di Medicina Interna, Endocrinologia E Diabetologia, Fondazione Policlinico Universitario Agostino Gemelli IRCCS Università Cattolica del Sacro Cuore Rome Italy; ^8^ Dermatologia, Dipartimento di Medicina e Chirurgia Traslazionale Università Cattolica del Sacro Cuore Rome Italy

**Keywords:** calcitonin gene‐related peptide, headache, idiopathic intracranial hypertension, papilledema, pseudotumor cerebri

## Abstract

**Objective:**

This study aimed to systematically review the literature on soluble biomarkers in adults with idiopathic intracranial hypertension (IIH).

**Background:**

Idiopathic intracranial hypertension is a multifactorial disorder marked by elevated intracranial pressure without a clear cause. Although it primarily affects overweight women of reproductive age, its pathogenesis remains incompletely understood. Symptoms include headache and visual disturbances due to papilledema. Increasing attention has focused on soluble biomarkers in serum and to understand disease mechanisms and aid diagnosis and management.

**Methods:**

This systematic review was conducted in accordance with Preferred Reporting Items for Systematic Reviews and Meta‐Analyses guidelines and registered in PROSPERO (CRD420250630653). The systematic literature search was conducted across PubMed, Web of Science, and Scopus for studies published in English between January 1995 and December 2024. Articles investigating cerebrospinal fluid (CSF) and/or blood biomarkers in adult patients with IIH were included. Multiple reviewers independently conducted screening, data extraction, and quality assessment. The risk of bias was evaluated using the ROBINS‐I tool.

**Results:**

A total of 38 studies on serum/plasma, urine, and CSF biomarkers met the inclusion criteria. The identified biomarkers were categorized into five main groups: (1) metabolic/endocrine, (2) systemic and neurogenic inflammation, (3) neurodegeneration, (4) neural antibodies and CSF dynamics, and (5) miscellaneous. Consistently elevated leptin levels were reported across studies, alongside evidence of cortisol dysregulation, altered androgen profiles, and insulin resistance. Inflammatory markers were frequently elevated, indicating a state of low‐grade systemic inflammation; however, findings regarding specific inflammatory markers were variable and lacked consistency. Two studies evaluated calcitonin gene‐related peptide (CGRP) in plasma, finding elevated levels in IIH, especially in those with migraine‐like headache. Neuronal markers like neurofilament light chain (NfL) were increased and correlated with disease severity. Emerging candidates included microRNAs, metabolites identified through metabolomic approaches, and novel proteins discovered via proteomic analyses.

**Conclusions:**

IIH appears to involve interplay of inflammatory, metabolic, and neurodegenerative processes. Although androgen dysregulation, leptin, and NfL show the most consistent association, substantial heterogeneity in study methods and populations limits generalizability. CGRP may emerge as a promising biomarker reflecting the predominant clinical symptom—headache—potentially guiding future therapeutic strategies.

## Plain Language Summary

Idiopathic intracranial hypertension (IIH) is disabling condition that causes elevated pressure around the brain, leading to symptoms such as headache and vision loss; little is understood about the biological causes of IIH. This study reviewed 38 studies of biomarkers found in blood, cerebrospinal fluid, and urine to shed light on the underlying biological mechanisms. The results suggest multiple pathways may be involved, including metabolic, inflammatory, and neurodegenerative processes, highlighting the need for further research to identify clinically useful diagnostic or prognostic biomarkers.

**Abbreviations**
11β‐HSD1: 11β‐hydroxysteroid dehydrogenase type 1
AQP: aquaporin
Aβ‐42: amyloid beta 42
BMI: body mass index
CCL: chemokine (C‐C motif) ligand
CGRP: calcitonin gene‐related peptide
CRP: C‐reactive protein
CSF: cerebrospinal fluid
GFAP: glial fibrillary acidic protein
GRADE: Grading of Recommendations, Assessment, Development and Evaluation
HGF: hepatocyte growth factor
ICP: intracranial pressure
Ig‐VH: immunoglobulin heavy chain variable region
IIH: idiopathic intracranial hypertension
IL: interleukin
MCP‐1: monocyte chemoattractant protein‐1
MOG: myelin oligodendrocyte glycoprotein
MRI: magnetic resonance imaging
NfL: neurofilament light chain
NGF: nerve growth factor
NLR: neutrophil‐to‐lymphocyte ratio
NSE: neuron‐specific enolase
NT‐proANP: N‐terminal pro A‐type natriuretic peptide
NT‐proBNP: N‐terminal pro B‐type natriuretic peptide
NT‐proCNP: N‐terminal pro C‐type natriuretic peptide
OCB: oligoclonal bands
PACAP: pituitary adenylate cyclase‐activating polypeptide (if used in your manuscript)
PAI‐1: plasminogen activator inhibitor‐1
PCOS: polycystic ovary syndrome
PICO: Population, Intervention, Comparison, Outcome
PLR: platelet‐to‐lymphocyte ratio
PRISMA: Preferred Reporting Items for Systematic Reviews and Meta‐Analyses
RAMP1: receptor activity‐modifying protein 1 (if relevant to your paper)
RBP: retinol‐binding protein
ROBINS‐I: Risk Of Bias In Non‐randomized Studies ‐ of Interventions
S1P: sphingosine 1‐phosphate
SiMoA: single molecule array
TNF‐α: tumor necrosis factor‐alpha

## INTRODUCTION

Idiopathic intracranial hypertension (IIH), also known as pseudotumor cerebri, is a neuro‐ophthalmic and metabolic disease characterized by increased intracranial pressure (ICP) without an identifiable causal factor.^1^ The two most prominent symptoms of IIH are progressive visual deterioration (loss of visual acuity and/or transient visual blurring) resulting from papilledema and headache. Additional manifestations include tinnitus, cranial nerve palsies, and cognitive deficits.^2^, ^3^

Idiopathic intracranial hypertension predominantly affects young women of childbearing age who are overweight and usually have androgen excess.^4^ The incidence of this pathology is increasing in line with escalating worldwide obesity rates.^5^

Brain magnetic resonance imaging (MRI) can reveal signs of increased ICP, including posterior globe flattening, empty sella turcica, distension of the optic nerve sheath, optic nerve tortuosity, optic nerve protrusion, and transverse sinus stenosis. However, the absence of these radiological findings does not rule out the diagnosis.^6^

Therapeutic options for this condition are limited, focusing mainly on reducing body weight and cerebrospinal fluid (CSF) production with carbonic anhydrase inhibitors, and neurosurgical approaches in selected cases or treatment refractory patients.^7^, ^8^

Despite treatment, the natural history of IIH is variable, with some patients resolving within months, whereas in other cases, the condition is relapsing or chronic.^9^

The etiopathogenesis of the disease has not been fully clarified. It has been suggested that leptin, cytokines, and steroid hormones secreted from adipocyte tissue may affect CSF production and drainage.^1^ Recent research has increasingly focused on the role of obesity‐related chronic inflammation in the development of the disease. Studies have shown elevated levels of several interleukins (ILs), including tumor necrosis factor‐α (TNF‐α), IL‐2, IL‐10, IL‐12, and IL‐17, highlighting the role of chronic inflammation in disease pathogenesis.^10^

Given the highly heterogeneous nature of disease course and symptoms, increasing focus is on identifying biomarkers in plasma or serum and CSF to enable precision medicine. These soluble biomarkers may provide valuable insights into the pathogenesis, course, and response to treatment of the disease. In addition to those mentioned above, inflammatory and metabolic markers, neurodegeneration markers, such as neurofilaments, markers implicated with altered CSF dynamics, and molecules related to symptoms like headache (e.g., calcitonin gene‐related peptide [CGRP]) have also been investigated.^11^

With the growing body of literature on various biomarkers across different domains, it remains challenging to identify the most promising biomarkers with potential for clinical implementation.

The aim of this systematic review is to collate the existing literature on soluble biomarkers in plasma and CSF in adults with IIH, thus providing clinicians with useful support in directing laboratory research.

## METHODS

### Search strategy and selection process

The systematic review was performed according to Preferred Reporting Items for Systematic Reviews and Meta‐Analyses (PRISMA) 2020 guidelines^12^ to investigate alterations in soluble biomarkers in patients with IIH. A comprehensive literature search of PubMed, Web of Science, and Scopus was performed for studies published in English from January 1995 to December 2024. The following keywords—including MeSH terms—were used to retrieve eligible articles: “biomarkers,” “immune,” “inflammation,” “pseudotumor cerebri,” and “idiopathic intracranial hypertension.” The detailed search strategy is available in the Supporting Information. This systematic review was registered and accepted in PROSPERO database with the following ID: CRD420250630653.

**FIGURE 1:**
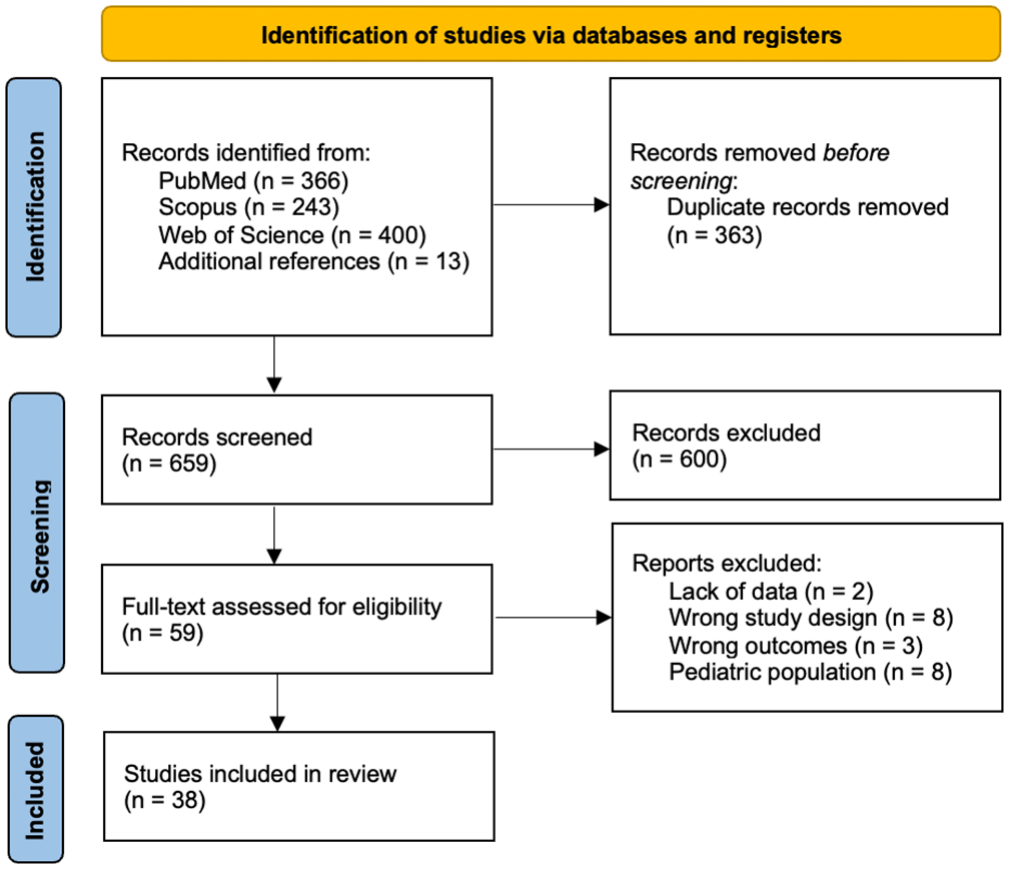
Preferred Reporting Items for Systematic Reviews and Meta‐Analyses flow‐chart. [Colour figure can be viewed at wileyonlinelibrary.com]

After searching the three databases, all results were collected. Duplicates were removed using Rayyan software.^13^ Three authors (F.Z., R.M., F.T.) independently screened titles and abstracts. Additional publications were retrieved from the reference list of relevant articles. Three reviewers (F.Z., R.M., A.F.) independently screened the full texts of selected articles, with a strong inter‐rater agreement (Cohen's κ 0.81 agreement; a senior author [M.R.] resolved discrepancies). A PRISMA flowchart depicting the selection and screening process is provided in Figure 1.

### Eligibility criteria

To meet the inclusion criteria, articles had to: (1) identify or examine soluble biomarkers in patients with IIH with or without papilledema; (2) be English peer‐reviewed articles published from January 1985 (year of publication of modified Dandy criteria by Smith et al.^14^) to December 2024; and (3) be original articles using qualitative, quantitative, or mixed methods. Exclusion criteria included: (1) gray literature (i.e., conference abstracts and dissertations), review articles, and case series with less than 10 patients; (2) animal‐based studies; and (3) studies conducted on the pediatric population.

### Data collection and criteria appraisal

Data extraction was performed from the text, tables, and figures of the included articles using the Population, Intervention, Comparison, Outcome (PICO) framework. The study population (P) included patients affected by IIH, as defined by the modified Dandy criteria,^14^ and/or the Revised Friedman criteria.^15^ The intervention (I) involved the assessment of soluble biomarkers; the comparison group (C) included healthy individuals or patients with other pathologies but without IIH. The primary outcome (O) to be extracted among studies was the evaluation of soluble biomarker/s in patients with IIH, and, when available, the comparison of these biomarkers between patients with IIH and healthy controls; the characterization of the study population, including clinical features, was also reviewed among selected studies. Additional data extracted included title, year of publication, study design, demographics of the study population, sample type, measurement methods, and main findings. Three investigators (F.Z., R.M., A.F.) conducted the data extraction, organizing the information into structured tables within Excel spreadsheets to facilitate descriptive analysis of each study. Subsequently, a senior investigator (M.R.) independently reviewed and double‐checked the extracted data from all articles. Any discrepancies between the investigators were resolved through discussion and consensus.

### Risk of bias and quality assessment

**FIGURE 2:**
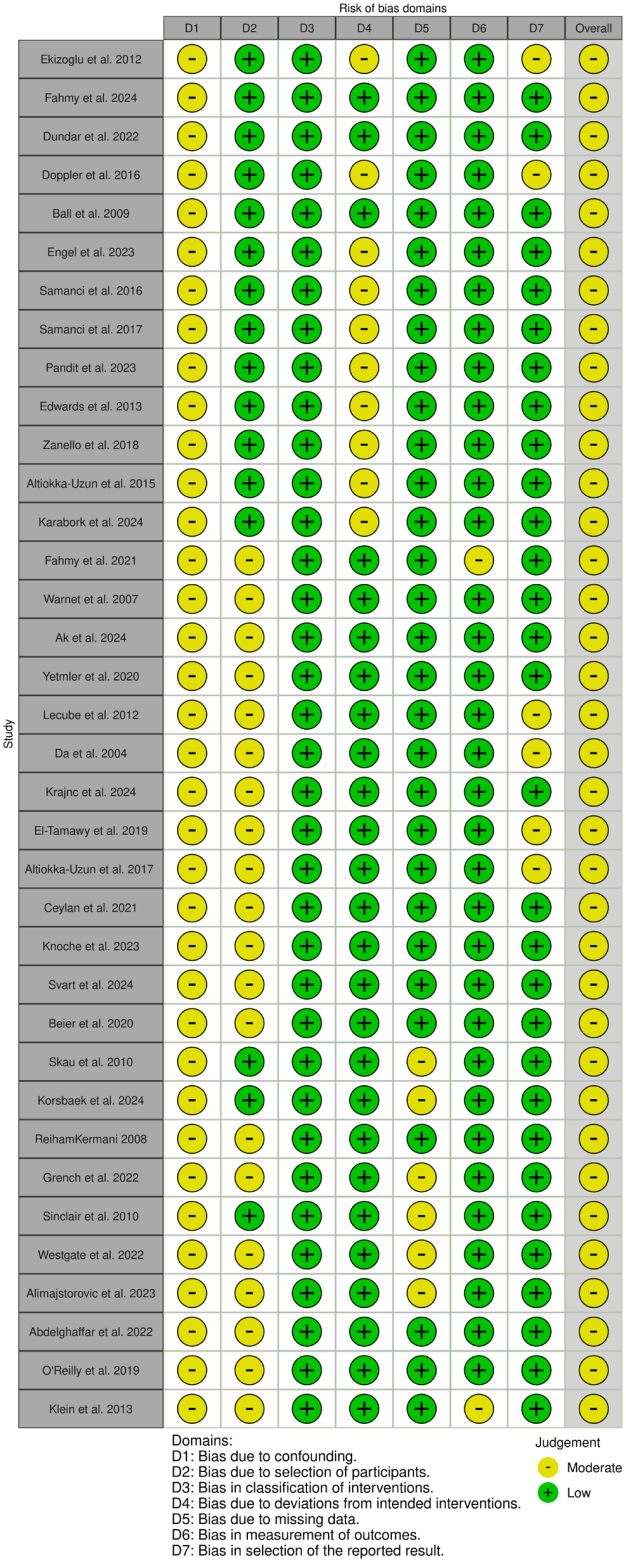
Risk of bias assessment across included studies using the ROBINS‐I tool. [Colour figure can be viewed at wileyonlinelibrary.com]

For all selected articles, we assessed the risk of bias and the quality of evidence using the ROBINS‐I tool^16^ and GRADE scale.^17^ Two authors independently performed the assessment, achieving a strong inter‐rater agreement (Cohen's κ 0.80 and 0.79 for risk of bias and quality, respectively). Any disagreements were solved through discussion and consensus. Overall, the risk of bias percentages for the studies ranged from 20% to 60%. All studies (*n* = 38) were judged to be at moderate risk of bias overall due to concerns primarily in the confounding domain (Figure 2).

### Data synthesis

A narrative synthesis was performed by grouping studies according to biomarker type. A quantitative meta‐analysis was not conducted due to substantial heterogeneity among the included studies due to the diversity of biomarkers analyzed and methodological variability in assays and study designs, which precluded meaningful pooling of results.

## RESULTS

The search of the literature yielded a total of 1009 results from three databases (PubMed, Scopus, and Web of Science) plus 13 additional references. Duplicate records were then removed (*n* = 363). A total of 659 articles were screened, and 600 records were excluded through title and abstract screening; 59 studies were considered relevant to our research question and were assessed for eligibility (Supporting Information). On full‐text review, 38 articles were included in the review on plasma/serum, CSF, and urinary biomarkers in patients with IIH, of which 35 were retrospective case–control studies, one prospective cohort study, and two cross‐sectional studies. Four main groups of relevant plasma/serum and/or urinary biomarkers and/or CSF biomarkers in patients with IIH were identified: (1) metabolic and endocrine, (2) systemic and neurogenic inflammation, (3) neurodegeneration, (4) neural antibodies and CSF dynamics, and (5) miscellaneous.

Studies could report biomarkers belonging to different groups, and the findings were discussed as appropriate in the corresponding paragraph. Detailed characteristics and main findings of each study are presented in Table 1.

**TABLE 1:** Soluble biomarkers in IIH.

First author (study design)	IIH/controls	Biomarkers tested	CSF levels	Blood/urinary levels	Comments	Biomarkers detection method
Metabolic and endocrine						
Ball et al. 2009 (case‐control)	26/62^a^	CSF and S: IL‐1β, IL‐6, IL‐8, TNF‐α, MCP‐1 (CCL2), HGF, NGF, insulin, leptin, resistin, adiponectin, PAI‐1 (active)	Leptin: ↑ IL‐8, MCP‐1, resistin, PAI‐1, HGF, IL‐6: no difference	Leptin: no difference Adiponectin: no difference IL‐8, MCP‐1, resistin, PAI‐1, TNF‐α, HGF: no difference		Multiplex immunoassays
Samanci et al. 2016 (case‐control)	39/40	S: IGF1, insulin, nesfatin, IL‐1B, IL‐6, IL‐8, leptin, PAI‐I, resistin, TNF‐α, MCP‐I		IL‐1β: ↑ IL‐8, TNF‐α: ↓		Multiplex immunoassays
Westgate et al. 2021 (case‐control)	97/43	CSF and S: leptin	Leptin: no difference	Leptin: ↑		Gene expression analysis
Abdelghaffar et al. 2022 (case‐control)	38/38	S: leptin, estradiol, testosterone, DHEA‐S CSF: leptin	Leptin: ↑	Leptin, estradiol, testosterone: ↑ DHEA‐S: no difference	IIH patients with BMI ≥30kg/m^2^ had significantly higher levels of serum leptin, CSF leptin, serum estradiol, serum testosterone	ELISA
Alimajstorovic et al. 2022 (case‐control)	60/20	CSF: formylpyruvate, maleylpyruvate/fumarylpyruvate, acetate S: formylpyruvate, riboflavin (vitamin B2) metabolites, pantetheine‐related metabolites, acylcarnitines, diacylglycerols, fatty acids, glycerophospholipids, lysoglycerophospholipids	Formylpyruvate: ↑ Riboflavin (vitamin B2) metabolites, pantetheine‐related metabolites, acylcarnitines, diacylglycerols, fatty acids, glycerophospholipids, lysoglycerophospholipids: altered	Formylpyruvate, maleylpyruvate/fumarylpyruvate: ↓ Acetate: ↑		Mass spectrometry
Klein et al. 2013 (cross‐sectional)	51	P: cortisol, testosterone, bioavailable testosterone, prolactin, DHEA‐S, androstenedione, insulin, estradiol, FSH, LH and aldosterone		Testosterone, DHEA‐S, androstenedione: ↑		Standard commercial assays
O'Reilly et al. 2019 (case–control)	70/100	S: testosterone, androstenedione, 11‐OH‐androstenedione, 11‐ketoandrostenedione, 5α‐reductase activity CSF: testosterone, androstenedione, DHEA, DHEA‐S, 11‐oxygenated androgens	Testosterone, androstenedione: ↑ DHEA‐S: ↓ 11‐oxygenated androgens: no difference	Testosterone, 5α‐reductase activity: ↑ Androstenedione, 11‐OH‐androstenedione, 11‐ketoandrostenedione: ↓		Mass spectrometry
Sinclair et al. 2010 (prospective cohort)	22	CSF and S: cortisol, cortisone, tetrahydrocortisol, 5α‐tetrahydrocortisol, tetrahydrocortisone, total cortisol metabolites, total cortisone metabolites, androsterone, etiocholanolone	CSF glucocorticoids: no change after therapeutic weight loss	Cortisol, cortisone, cortisol/cortisone ratio: no change Tetrahydrocortisol +5α‐tetrahydrocortisol/tetrahydrocortisone, total cortisol/cortisone metabolites, androsterone, androsterone/etiocholanolone ratio: ↓	Weight loss led to ↓ in 11β‐HSD1 activity, correlated with ↓ in intracranial pressure; ↓ in CSF cortisone correlated with ↓ weight	Gas chromatography/mass spectrometry and liquid chromatography/tandem mass spectrometry for CSF and serum
Westgate et al. 2022 (case–control)	27/17	U: 11β‐HSD1 activity, cortisol, 5α‐reductase activity, pregnanediol		U: 11β‐HSD1 activity, cortisol, 5α‐reductase activity, pregnanediol: ↑		Gas chromatography/mass spectrometry; liquid chromatography/mass spectrometry
Grech et al. 2022 (case–control)	84/20	23 CSF, 12 S, 9 U metabolites	Acetate, lactate:pyruvate ratio: ↑ Lactate, pyruvate, fumarate, urea: ↓ Altered ketones (3‐HB, acetoacetate)	S: lactate:pyruvate ratio: ↑; pyruvate: ↓ U: urea, citrate: ↓	Weight loss over 12 months led to partial or full normalization of several key metabolites including acetate, L:P ratio, urea, and ketones	^1^H‐NMR spectroscopy, comprehensive untargeted metabolomics
Systemic and neurogenic inflammation						
Ceylan et al. 2021 (case–control)	33/33	CSF: NLR and PLR	NLR, PLR: ↑			
Dhungana et al. 2009 (case–control)	8/8	CSF and S: 42 different cytokines, chemokines, growth factors (chemokine CCL2, IL‐1a, leptin)	CCL2: ↑	CCL7, CCL8, IL‐1a, leptin: ↑ IL‐1a: no difference		Cytokine antibody array, ELISA
Fahmy et al. 2024 (case–control)	36/36	S: LDH, CRP, NLR, PLR		LDH, CRP, NLR, PLR: ↑		ELISA
ReihamKermani et al. 2008 (case–control)	14/14	CSF: IL‐6, Il‐10	IL‐6: ↑			ELISA
Edwards et al. 2013 (case–control)	17/53^a^	CSF and S: IL‐1β, IL‐2, IL‐4, IL‐6, IL‐8, IL‐10, IL‐12p70, IL‐17, IL‐22, IL‐23, IFN‐γ, TNF‐α, TGF‐β, osteopontin	IL‐17, IL‐2: ↑		IL‐2, IL‐8, and IL‐17 were ↑ in CSF than serum; IL‐1β, IL‐4, IL‐22, IFN‐γ, and TNF‐α were ↑ in serum than CSF	Multiplex immunoassays
Altiokka‐Uzun et al. 2015 (case–control)	26/33^a^	CSF and S: IL‐4, IL‐10, IL‐12, IL‐17, TNF‐α, IFN‐γ CSF: OCBs	OCBs detected in 8 (30.77%) All cytokines (except TNF‐α): ↑	TNF‐α, IFN‐γ, IL‐4, IL‐10, IL‐12, IL‐17: ↑	IL	ELISA
Karabork et al. 2024 (case–control)	16/22^a^	CSF: TNF‐α, IL‐6, AP‐1			AP‐1 potential biomarker for rapid diagnosis and useful for distinguishing IIH from MS	ELISA
Fahmy et al. 2021 (case–control)	36/30	S: TNF‐α		TNF‐α: ↑	TNF‐α: negatively correlated with perimetry grade, CSF opening pressure	ELISA
Ak et al. 2024 (case–control)	36/36	S: CGRP		CGRP: ↑	Chronic migraine and IIH patients had ↑ levels of CGRP compared to controls	ELISA
Da et al. 2004 (case–control)	10	CSF: Ig heavy chain variable region (VH) genes of B cells	Polyclonal B‐cell expansion in 6/10 IIH patients			Gene expression analysis
Krajnc et al. 2024 (case–control)	26/87	S: CGRP		CGRP: ↑	CGRP was higher in IIH patients with migraine‐like headache vs. non‐migraine–like headache and headache absence	ELISA
El‐Tamawy et al. 2019 (case–control)	27/21	S: IL‐4, IL‐10, TNF‐α CSF: OCBs	OCBs: detected in 6 (22%)	IL‐4, IL‐10, TNF‐α: ↑		ELISA
Altiokka‐Uzun et al. 2017 (casa‐control)	34/40	S: NLR, PLR		NLR, PLR: ↑		CBA
Neural antibodies and CSF dynamics						
Ekizoglu et al. 2012 (case–control)	29/30	S: Ab anti‐AQP4		Ab anti‐AQP4: not detected		Immunofluorescence detection kit, indirect immunohistochemistry test
Doppler et al. 2016 (case–control)	28/29	CSF and P: AQP1, AQP4, leptin, RBP‐4	AQP4: ↓ Leptin: ↑ AQP1, RBP‐4: no difference	AQP4, leptin: ↑ AQP1, RBP‐4: no difference	Leptin in CSF and plasma correlated with weight, BMI, and body fat	ELISA
Yetimler et al. 2020 (cross‐sectional)	58	CSF and S: Ab anti‐GFAP	Ab anti‐GFAP: not detected	Ab anti‐GFAP: detected		CBA
Neurodegeneration						
Engel et al. 2023 (case–control)	87/93	CSF and S: NfL, GFAP	GFAP: ↑	GFAP: no difference		SiMoA
Samanci et al. 2017 (case–control)	36/40	S: NSE		NSE: no difference		ELISA
Knoche et al. 2023 (case–control)	35/12	CSF: NfL	NfL: ↑			NA
Svart et al. 2024 (case–control)	37/35	CSF: NfL, Aβ‐42, T‐tau, P‐tau S: NfL	NfL, total‐tau/Aβ‐42: ↑			ECLIA, SiMoa
Beier et al. 2020 (cross‐sectional)	61	CSF and S: NfL			NfL ↑ in moderate/severe papilledema vs. minor/no papilledema	SiMoA
Miscellaneous						
Lecube et al. 2012 (case–control)	8/8	CSF: osteopontin, metallothionein‐1E, neurosecretory protein VGF, neuroendocrine protein 7B2, chromogranin‐A, secretogranin‐1 precursor, fibrinogen c chain, testican‐1, fibrinogen b chain, isoform 10 of fibronectin, metallothionein‐3, alacid glycoprotein 2, haptoglobin, ProSAAS, autotaxin, prostaglandin‐H2, D‐isomerase, metallopeptidase inhibitor type 2, galectin‐3 binding protein	Proteins ↑ in the CSF of obese patients compared to non‐obese subjects: osteopontin, metallothionein‐1E, neurosecretory protein VGF, neuroendocrine protein 7B2, chromogranin‐A, secretogranin‐1 precursor, fibrinogen c chain, testican‐1, fibrinogen b chain, isoform 10 of fibronectin, metallothionein‐3, a1‐acid glycoprotein 2 and haptoglobin			Mass spectrometry, Western blot, ELISA
Dundar et al. 2022 (case–control)	24/21	CSF: sortilin‐1, lipocalin‐2, autotaxin, decorin, IL‐33	Lipocalin‐2, sortilin‐1, IL‐33, autotaxin: ↑ Decorin: ↓			ELISA
Korsbaek et al. 2024 (case–control)	60/35	S: SIP1, adenosine, glutamate; CSF: LysoPC18, LysoPC16	LysoPC‐18, LysoPC‐16: ↓	S1P, adenosine, glutamate: ↓		Mass spectrometry
Zanello et al. 2018 (case–control)	22	CSF and P: miRNA	miR‐9, miR‐16: ↑	miR‐9, miR‐16: ↑		TaqMan OpenArray miRNA
Skau et al. 2010 (case–control)	40/20	CSF and S: NT‐proANP, NT‐proCNP P: NT‐proBNP	NT‐proANP, NT‐proCNP: no difference	NT‐proCNP and NT‐proBNP: ↓		In‐house processing‐independent assays
Pandit et al. 2023 (case‐control)	13/20	CSF: 232 proteins through proteomics	37 proteins differentially expressed in IIH		Neurosecretory, neuroendocrine, and inflammatory proteins involved in IIH	Mass spectrometry
Warner et al. 2007 (case‐control)	28/59	CSF and S: RBP, retinol, retinol/RBP ratio	Retinol: ↑ RBP: ↓	Retinol: ↑ RBP: no difference		NANORID RID kit

*Note*: The table summarizes all biomarker studies in IIH resulting from the systematic review.

Abbreviations: Ab, antibodies; Aβ‐42, amyloid beta‐42; AQP, aquaporin; CBA, cell‐based assay; CCL2, chemokine (C–C motif) ligand 2; CCL7, chemokine (C–C motif) ligand 7; CCL8, chemokine (C–C motif) ligand 8; CGRP, calcitonin gene‐related peptide; CRP, C‐reactive protein; CSF, cerebrospinal fluid; DHEA, dehydroepiandrosterone; DHEA‐S, dehydroepiandrosterone sulfate; ECLIA, electrochemiluminescence immunoassay; ELISA, enzyme‐linked immunosorbent assays; FSH, follicle‐stimulating hormone; GFAP, glial fibrillary acidic protein; HGF, hepatocyte growth factor; IFN‐γ, interferon gamma; IIH, idiopathic intracranial hypertension; IL, interleukin; LDH, lactate dehydrogenase; LH, luteinizing hormone; MCP‐1, monocyte chemoattractant protein‐1; miR, microRNA; NfL, neurofilament light chain; NLR, neutrophil‐to‐lymphocyte ratio; NSE, neuron‐specific enolase; OCBs, oligoclonal bands; P, plasma; PAI‐1, plasminogen activator inhibitor‐1; PLR, platelet‐to‐lymphocyte ratio; proBNP, pro‐B–type natriuretic peptide; proCNP, pro‐C–type natriuretic peptide; RBP, retinol‐binding protein; RID, radial immunodiffusion; S, serum; SiMOA, single molecule array; T‐tau, total tau protein; TNF‐α, tumor necrosis factor alpha; U, urinary.

^a^ Comparison groups consisting of individuals with multiple sclerosis or other inflammatory conditions.

### Metabolic and endocrine

Metabolic and endocrine plasma/serum, CSF, and urinary biomarkers were evaluated in 10 different studies.

Given the association between obesity and IIH, attention has turned to adipose tissue as an active endocrine organ that secretes a range of bioactive molecules, including proinflammatory cytokines, chemokines, and hormones. Among the most prominent are the adipokines leptin and adiponectin.^18^ Leptin, a pro‐inflammatory peptide hormone, regulates satiety via the hypothalamus and crosses the blood–brain barrier. In contrast, adiponectin exerts anti‐inflammatory effects and enhances insulin sensitivity.^18^ Several studies have investigated adipokine levels in serum and CSF in patients with IIH.^19^, ^20^, ^21^, ^22^, ^23^

Doppler et al.^19^ found that CSF and serum leptin levels were elevated in 28 overweight patients with IIH (25 females) compared with age and sex‐matched non‐overweight controls. Leptin levels were correlated with patients' weight, body mass index (BMI), and body fat.^19^ Two other studies confirmed these findings by assessing leptin levels in serum and CSF of patients with IIH and healthy controls, resulting in significantly higher concentration levels in the affected group.^20^, ^21^ Notably, in the aforementioned study by Ball et el.,^20^ the group labeled as “healthy controls” included patients with multiple sclerosis and other unspecified neurological conditions.

Similarly, Westgate et al.^22^ found elevated fasted serum leptin in female patients with IIH compared with female weight‐matched controls; however, they reported no differences in CSF and serum/CSF ratio between patients with IIH and controls. Westgate et al.^22^ also found significantly higher levels of fasting insulin compared to controls and markers of insulin resistance, concluding that IIH can be considered a metabolic disorder in which adipose tissue dysfunction is a feature of the disease.^22^

However, other studies gave inconsistent results; for example, Samanci et al.^23^ found no differences between the adipokine serum levels (leptin, adiponectin, and nefastin) between patients with IIH and BMI‐matched controls. Furthermore, there were no correlations between adipokine levels and age, BMI, and disease duration. No significant differences in adipokine levels between patients with IIH regarding visual deterioration emerged.^23^ It is crucial to notice that, in this study, all patients were in remission and under dietary adjustments, possibly affecting the adipokine status.

Obesity is also linked with excess androgen production. Given this, androgen dysregulation has been investigated in IIH. We identified three relevant studies on the topic.^4^, ^24^, ^25^

Increased serum and CSF testosterone levels have also been reported in IIH compared to controls. A study reported that 38 IIH female patients had significantly higher levels of serum testosterone, serum leptin, CSF leptin, and serum estradiol than female controls. Furthermore, all these hormones were significantly elevated in patients with high BMI.^24^

One case–control study comparing 70 patients with IIH to age‐, sex‐, and BMI‐matched individuals (*n* = 40) with obesity or polycystic ovarian syndrome (PCOS) (*n* = 60) reported elevated serum testosterone and reduced androstenedione in the IIH group. In the same study, urinary steroid excretion in a subsample of patients with IIH was evaluated, finding increased 5α‐reductase activity in comparison to patients with PCOS and obese controls.^4^

In a study by Klein et al.^25^ involving 51 female patients with IIH, hyperandrogenism was associated with a younger age at disease onset but showed no correlation with BMI or disease duration. However, the absence of a control group and the assessment of patients at varying stages of disease and treatment introduce substantial heterogeneity, limiting the interpretation of the findings.^25^

Another group of studies investigated cortisol metabolism, with a particular focus on 11β‐hydroxysteroid dehydrogenase type 1 (11β‐HSD1), an enzyme that plays a key role in regulating local cortisol availability and is implicated in the pathogenesis of obesity. In patients with IIH, biomarkers measured in plasma, CSF, and urine indicated increased systemic activity of 11β‐HSD. Two studies demonstrated that weight loss was achieved through bariatric surgery or a low‐calorie diet, significantly reducing systemic activity of the enzyme.^26^, ^27^ Notably, the reduction in 11β‐HSD1 activity correlated with decreased intracranial pressure, suggesting a mechanistic link between cortisol metabolism and IIH pathophysiology.^26^ The two aforementioned studies lack a control group.^26^, ^27^

### Systemic and neurogenic inflammation

Systemic and neurogenic inflammation biomarkers were evaluated in 13 studies, of which six considered blood biomarkers, four considered CSF biomarkers, and five considered both.

Among the serum studies, one compared serum lactate dehydrogenase (an important enzyme of the anaerobic metabolic pathway), C‐reactive protein (CRP), neutrophil‐to‐lymphocyte ratio (NLR), and platelet‐to‐lymphocyte ratio (PLR) in patients with IIH and healthy controls, finding that they were significantly higher in affected patients.^28^ Notably, there was a significant positive correlation between serum CRP and the presence of venous stenosis in magnetic resonance venography.^29^

These data were also confirmed by Ceylan et al.,^30^ who found that serum NLR and PLR levels were significantly higher in patients with IIH than in controls. Fahmy et al.^29^ also measured TNF‐α levels in patients with IIH. They examined its relationship with ophthalmological parameters and CSF opening pressure, finding that serum TNF‐α levels were significantly higher in patients with IIH than in healthy controls and significantly negatively correlated with the grade of perimetry and CSF opening pressure. These findings contrast with the study conducted by Samanci et al.^23^ in which TNF‐α and IL‐8 levels were significantly lower in the IIH group compared to the control group; in the same study, IL‐1β level was found to be significantly higher.

Considering the CSF studies group, a study evaluated IL‐6 and IL‐10 CSF concentration levels, finding that only IL‐6 was significantly higher in patients with IIH than in controls. Dundar et al.^31^ focused on IL‐33 CSF concentration, which was significantly increased in patients with IIH.

Karabork et al.^32^ evaluated CSF levels of activator protein‐1 (AP‐1), TNF‐α, and IL‐6 in patients with IIH compared to 22 people with multiple sclerosis. There was no difference between the groups in CSF TNF‐α and IL‐6 levels. However, AP‐1 concentration was significantly higher in the IIH group, suggesting it could be a supportive parameter in differentiating both diseases.^32^

Some authors have evaluated both serum and CSF inflammatory biomarkers in patients with IIH. Among these, one study investigated the presence of the following markers: IL‐1β, IL‐6, IL‐8, TNF‐α, monocyte chemoattractant protein‐1 (MCP‐1), hepatocyte growth factor (HGF), nerve growth factor (NGF), insulin, leptin, resistin, adiponectin, and plasminogen activator inhibitor 1 (PAI‐1).^20^ Serum IL‐1β, IL‐6, and CSF TNF‐α were below the sensitivity of the assays. No significant differences were observed between individuals with IIH and controls in terms of serum IL‐8, MCP‐1, resistin, PAI‐1, TNF‐α, HGF, adiponectin, or CSF levels of IL‐8, MCP‐1, resistin, PAI‐1, HGF, and IL‐6.^20^

Two studies evaluated serum and CSF interleukin levels in patients with IIH, using comparison groups consisting of individuals with multiple sclerosis or other inflammatory conditions rather than healthy controls. Although most of the cytokines measured were significantly elevated in the IIH group, this choice of control group limits the interpretation of the findings.^10^, ^33^

Two studies have also investigated the correlation between cytokines and OCB. The first study found that serum levels of TNF‐α, IL‐4, and IL‐10 were significantly higher in patients with IIH than in healthy controls, and TNF‐α levels were significantly higher in OCB‐positive patients compared to OCB‐negative ones.^34^ The other study confirmed elevated levels of TNF‐α and IFN‐γ in OCB‐positive patients; h owever, overall CSF cytokine levels were comparable between OCB‐positive and OCB‐negative individuals.^33^

Dhungana et al.^21^ evaluated serum and CSF concentration levels of 42 different cytokines, finding elevated levels of CCL2 in the CSF and serum IL‐1α, CCL7, and CCL8 levels in patients with IIH compared with the control group, with significantly elevated CSF CCL2 in patients with IIH.

Da et al.^35^ investigated immunoglobulin heavy chain variable region (Ig‐VH) genes of B cells in the CSF of 10 patients with IIH, with six of 10 patients showing polyclonal B‐cell expansion. Nucleotide sequences of intrathecal Ig‐VH genes revealed both T‐cell–dependent and T‐cell–independent B‐cell expansion in the CSF, and an elevation of immunoglobulin G (IgG) was correlated to intrathecal B‐cell expansion, suggesting that B‐cell–mediated immune response might play a role in the pathogenesis of IIH.^35^

Finally, markers associated with neurogenic inflammation, particularly linked to migraine pathogenesis, have been investigated.

Kranjc et al.^11^ conducted a longitudinal pilot study including patients with IIH, episodic migraine in the interictal phase, and healthy controls, finding that within patients with IIH, those with migraine‐like headache had significantly higher plasma CGRP levels assessed with ELISA commercial kits (for α‐ and β‐CGRP) compared to those with non‐migraine–like headache and headache absence. Serum CGRP levels were also assessed in another study with the same methodology, which confirmed significantly increased levels in IIH and patients with chronic migraine compared to the healthy controls.^36^ In the prospective part of this study, patients with ongoing headache at a 6‐month evaluation had significantly higher values of serum CGRP, indicating a strict correlation between this symptom and CGRP in IIH.^36^

### Neurodegeneration

Neurodegeneration biomarkers were assessed in five studies.

Samanci et al.^37^ assessed serum concentration levels of neuron‐specific enolase (NSE), expressed mainly by neuronal cells, which is a long‐standing marker for neuronal damage. In this study, no significant difference was found between IIH and healthy controls and different clinical subgroups of IIH.

Knoche et al.^38^ and Svart et al.^39^ evaluated CSF neurofilament light chain (NfL) concentrations, a marker of axonal damage, in patients with IIH and healthy controls, finding significantly higher levels of NfL in patients with IIH. The latter study also described higher plasma NfL and CSF total‐tau and amyloid‐beta 42 (Aβ‐42) levels in the affected group.^39^ Beier et al.^40^ also focused on NfL concentration levels in CSF and serum of patients with IIH. Interestingly, the average CSF NfL levels in patients with moderate and severe papilledema were increased compared to the ones with minor and absence of papilledema.^40^ The correlation between elevated serum and CSF levels of NfL and IIH was further confirmed by another study by Engel et al.^41^ This study also measured glial fibrillary acidic protein (GFAP)—a class III intermediate filament protein contributing to the mechanical support of astrocytes and ependymal cells—concentrations using single molecule array technology, finding no significant differences between GFAP levels in patients with IIH and healthy controls.^41^

### Neural antibodies and CSF dynamics

Neural antibodies and CSF dynamics markers, in particular glial ones, were assessed in three studies.

Aquaporin 1 (AQP1), expressed in epithelial cells of the choroid plexus, and aquaporin 4 (AQP4), present in ependymal cells and glia limitants, have been proposed to play a significant role in CSF production and homeostasis.^42^ Anti‐AQP4 antibodies are associated with neuromyelitis optica spectrum disorder; antibodies against myelin oligodendrocyte glycoprotein (MOG) are found in a similar demyelinating disorder.^43^

Ekizoglu et al.^44^ investigated the presence of AQP4 water channel antibodies in 29 patients with IIH, of which no one tested positive. Different results are reported in another study that assessed AQP1 and AQP4 CSF and serum levels in affected patients and healthy controls. In patients with IIH, the AQP4 CSF to plasma ratio was reduced compared to controls, and the AQP4 CSF levels were decreased, whereas plasma levels were slightly increased in the IIH group.^19^ No difference in the AQP1 CSF or plasma levels emerged between the patients and controls.^19^

Altiokka‐Uzun et al.^45^ investigated the presence of antibodies against AQP4 and anti‐MOG, and uncharacterized neuronal membrane antigens: none of the patients with IIH showed MOG and AQP4 antibodies; however, serum IgG of five patients with IIH exhibited reactivity against membrane antigens of rat hippocampal and cortical neurons. Intriguingly, among these five patients, three had nonspecific white matter lesions on the brain MRI.

One study evaluated antibodies against GFAP in patients with IIH. Yetimler et al.^46^ evaluated the presence of anti‐GFAP antibodies in serum and CSF of affected patients: even though no positivity in CSF samples was found, two positive serum samples were found from affected patients with good responses to treatment. Subsequent controls in one of these showed decreased antibody levels during remission.^46^

### Miscellaneous

This section includes seven studies. Two studies employed proteomic analyses of CSF, assessing 348 and 233 proteins, respectively. The first study identified differences in CSF protein expression related to inflammatory, endocrine, and neuroplasticity pathways when comparing obese and non‐obese individuals.^47^ The second study, which included a control group, identified 37 proteins that were differentially expressed between groups, 24 upregulated and 13 downregulated. Additionally, two proteins were found to differ between overweight and non‐overweight individuals within the IIH cohort.^48^ However, the findings from both studies are notably limited by small sample sizes.^47^, ^48^

Two studies investigated the metabolomic profile of patients with IIH.^49^, ^50^ The first involved 66 patients with IIH identified significant perturbations in multiple amino acid metabolic pathways and lipid classes in both serum and CSF, when compared to BMI‐ and sex‐matched controls. Notably, serum lipid metabolites were significantly elevated in IIH. Although in CSF, formylpyruvate and maleylpyruvate/fumarylpyruvate were found to be reduced at baseline but returned to control levels following a 12‐month weight loss intervention.^49^

The second study evaluated metabolites in serum, urine, and CSF, including ketone bodies, which were normalized after therapeutic weight loss.^50^

Other studies have investigated a diverse range of proteins, lipid mediators, and neurotransmitters involved in inflammatory pathways, neural plasticity, CSF dynamics, and vascular tone.^31^, ^51^

Among these, sphingosine 1‐phosphate (S1P) has emerged as a promising marker. S1P is a pleiotropic lipid mediator involved in key cellular processes such as proliferation, cytoskeletal rearrangement, adhesion, and immune modulation. It is expressed in the cerebral vasculature and choroid plexus, where it may contribute to the regulation of BBB integrity. Notably, S1P levels were found to be reduced in patients with IIH and were associated with subsequent optic nerve atrophy in one study evaluating new‐onset IIH, suggesting a potential prognostic role.^51^

Based on the hypothesis that free retinol (a vitamin A precursor) may be toxic to arachnoid villi and inhibit CSF resorption, Warner et al.^52^ examined serum concentrations of retinol and retinol‐binding protein (RBP) in individuals with and without IIH. They found that affected patients had higher retinol levels in both CSF and serum, whereas CSF RBP levels were lower in the IIH group. In both serum and CSF, patients with IIH had the highest retinol/RBP ratio, indicating an increased presence of unbound retinol in their CSF.^52^

Zanello et al.^53^ reported interesting results, conducting preliminary research on the molecular pathways associated with the elevation of ICP in IIH. They found an upregulation of miR‐9 and miR‐16 in the CSF and plasma of patients with IIH.^53^

Skau et al.^54^ compared serum levels of NT‐proBNP, NT‐proANP, and NT‐proCNP and CSF levels of NT‐proANP and NT‐proCNP in patients with IIH and healthy controls. No differences in peptide concentrations between patients and controls were observed in the CSF. Plasma proCNP and proBNP were lower in patients with IIH compared with controls.^54^

## DISCUSSION

This review highlighted the multifaceted nature of IIH, where metabolic, hormonal, and inflammatory factors interact in a complex pathophysiological network. Obesity emerges as a central contributor to the pathogenesis of IIH, driving systemic low‐grade inflammation and dysfunction of the adipose tissue. The endocrine activity of adipose tissue appears particularly important in IIH, with growing evidence supporting its role in ICP. Leptin consistently stands out among the metabolic biomarkers.^19^, ^20^, ^21^, ^22^, ^23^ Several studies have reported elevated leptin levels in the serum and CSF of individuals with IIH.^22^ Insulin resistance observed in individuals with IIH also stresses the concept of IIH as a metabolic disorder.^22^

The role of androgen dysregulation has also drawn attention, with elevated testosterone levels found in individuals with IIH.^4^, ^24^, ^25^ Notably, IIH appears to have a distinct metabolic‐hormonal profile from diseases such as PCOS despite overlapping features like obesity, hyperandrogenism, and insulin resistance.^4^

Cortisol metabolism, particularly via 11β‐HSD1, represents another promising but largely underexplored pathway. Increased enzyme activity has been linked to IIH pathogenesis and shown to normalize following weight loss, with corresponding decreases in ICP.^26^, ^27^

In parallel, numerous studies assessing inflammatory biomarkers highlight a clear pro‐inflammatory background in IIH, with elevated CRP, NLR, PLR, and several cytokines.^30^, ^31^ Although consistent findings on the alteration of a specific inflammatory marker remain inconsistent, the overall evidence supports the presence of altered inflammatory signaling skewed toward a proinflammatory state.^10^

Finally, regarding neurodegeneration markers, NfL seems the most promising. It has been found elevated in both CSF and serum of patients with IIH compared to healthy controls.^38^, ^39^ Importantly, CSF NfL levels seem correlated with markers of disease severity, such as increased ICP, papilledema, and optic nerve damage, supporting its potential utility as a prognostic biomarker for visual outcomes and structural neuronal injury.^40^

Moreover, there is a growing interest in the evaluation of other neurodegenerative markers in individuals with IIH, such as NSE, total tau, and Aβ‐42, which have produced less consistent results.^37^, ^46^

The hypothesis that the immune system may be involved remains limited, particularly regarding the detection of altered levels of autoantibodies in a subset of patients which could potentially influence CSF dynamics, such as those targeting AQP4.^45^

Another interesting factor is the potential role of CGRP, a key molecule implicated in the pathogenesis of migraine, especially among those with IIH who experience a migraine‐like headache.^11^, ^36^, ^55^ This finding suggests a possible overlap of pathogenic mechanisms in the two conditions, opening potential avenues for shared therapeutic management in light of the introduction of novel treatments targeting CGRP for acute migraine and preventive treatment.^56^

In this context, a case series of seven patients with IIH reported successful treatment of refractory chronic migraine‐like headache using anti‐CGRP monoclonal antibodies, specifically in individuals whose papilledema had resolved.^57^ The authors suggested that CGRP may play a mechanistic role in mediating elevated ICP‐related headache in active IIH due to pathological CGRP release from the richly trigeminovascular‐innervated dural sinuses, which may become congested in the setting of raised ICP.^57^, ^58^ The therapeutic potential of CGRP monoclonal antibodies in managing IIH‐associated headache deserves further investigation in controlled clinical trials.

However, it is important to notice that CGRP in plasma is difficult to evaluate due to its very low concentrations and short half‐life. Moreover, commercially available ELISA kits often have low sensitivity and do not distinguish between the α‐ and β‐CGRP isoforms, further complicating the interpretation of results.^7^

The current literature also includes a variety of novel and miscellaneous biomarkers such as microRNAs (especially miR‐9 and miR‐16), and proteomic findings illustrate a complex molecular landscape that needs to be clarified with larger studies.^49^, ^50^ Proteomic and lipidomic profiling has similarly revealed perturbations in pathways implicated in immune regulation, neural signaling, and CSF dynamics.^49^, ^50^ These cumulative data suggest that integrative multi‐omic approaches may be essential for untangling the molecular complexity of IIH and may ultimately facilitate the identification of robust, clinically actionable biomarker profiles for diagnosis, risk stratification, and therapeutic monitoring.

### Limitations

This review has several limitations that should be acknowledged. There is a substantial heterogeneity across studies regarding patient populations and control groups. The study population comprised patients with IIH with different disease duration and severity, including both medically and surgically treated and untreated individuals. The control population was not always weight‐matched, and several studies considered a control population with other neurological conditions.

Variability in detection methods for biomarkers further complicates a possible direct comparison. Additionally, important clinical variables such as ICP levels, presence or absence of papilledema, and comorbid conditions were inconsistently reported, limiting the ability to draw conclusions about their reliability as biomarkers.

## CONCLUSIONS

Our review points to IIH as a disorder in which inflammatory, metabolic, and neurodegenerative pathways converge. Yet the wide range of study populations, technical methods, and clinical presentation, along with confounding variables such as obesity, complicates interpretation. These factors underline the need for large, prospective, and methodologically uniform studies to delineate causal versus associative biomarkers, determine diagnostic thresholds, and pinpoint clinically actionable measures. Moving forward, it will be essential to shift from isolated biomarker toward the development of a disease‐specific molecular signature. In this context, omics platforms, particularly metabolomics and proteomics, offer a promising framework for capturing the complex biology of IIH and advancing precision diagnostics and targeted therapeutic strategies.

## AUTHOR CONTRIBUTIONS

**Marina Romozzi:** Conceptualization; data curation; formal analysis; investigation; methodology; project administration; supervision; validation; visualization; writing – original draft; writing – review and editing. **Fabio Zeoli:** Conceptualization; data curation; formal analysis; investigation; methodology; resources; software; writing – original draft; writing – review and editing. **Renata Martinelli:** Data curation; formal analysis; investigation; methodology; writing – original draft. **Federico Tosto:** Investigation; methodology; project administration; visualization; writing – original draft. **Antonio Funcis:** Investigation; methodology; writing – original draft. **Giuseppe Garignano:** Conceptualization; methodology. **Sabrina Chiloiro:** Conceptualization; investigation. **Lucia Di Nardo:** Supervision; validation; visualization. **Catello Vollono:** Conceptualization; investigation; methodology; project administration; supervision; validation; visualization. **Alessandro Olivi:** Supervision; visualization. **Paolo Calabresi:** Conceptualization; methodology; project administration; supervision; validation; visualization; writing – original draft. **Francesco Signorelli:** Conceptualization; methodology; project administration; resources; supervision; validation; visualization; writing – original draft; writing – review and editing.

## FUNDING INFORMATION

This research received no specific grants from funding agencies in the public, commercial, or not‐for‐profit sectors.

## CONFLICT OF INTEREST STATEMENT

**Marina Romozzi**, **Fabio Zeoli**, **Renata Martinelli**, **Federico Tosto**, **Antonio Funcis**, **Giuseppe Garignano**, **Sabrina Chiloiro**, **Lucia Di Nardo**, **Catello Vollono**, **Alessandro Olivi**, **Paolo Calabresi**, and **Francesco Signorelli** declare no conflicts of interest.

## Supporting information


Table S1.

## Data Availability

The data collected for the review are available from the corresponding author on reasonable request.
