# Supplementary material for: Cerebrospinal fluid and serum biomarkers in idiopathic intracranial hypertension: A systematic review
Source: Headache. 2025 Aug 8;65(8):1462–76. doi: 10.1111/head.15023 (PMC12455426; doi:10.1111/head.15023)
Supplement: Supplementary file 1 — Table S1. [file HEAD-65-1462-s001.docx]

**Soluble Biomarkers in Idiopathic Intracranial Hypertension: a Systematic Review**

***Supplementary Material***

**Search strategy**

*Filters*: English, 1985- December 2024, Humans

- PubMed: (((((((biomarkers[MeSH Terms]) OR (Biomarker*)) OR ("immunological"[All Fields])) OR (immune)) OR (Immun*)) OR (Inflammation[MeSH Terms])) OR (inflammatory)) AND (((pseudotumor cerebri[MeSH Terms]) OR ("pseudotumour cerebri"[All Fields])) OR (Idiopathic intracranial hypertension)) → **366 hits**
- Scopus: TITLE-ABS-KEY ( "idiopathic intracranial hypertension" ) AND TITLE-ABS-KEY ( biomarker* OR inflammat* ) → **243 hits**
- Web of Sciences: (((ALL=(biomarker*)) OR ALL=(immun*)) OR ALL=(inflammatory)) OR ALL=(inflammation) AND ((ALL=(idiopathic intracranial hypertension)) OR ALL=("Pseudotumour cerebri")) OR ALL=("Pseudotumor cerebri") **→ 400 hits**
- Additional articles from reference lists**: 13 hits**

**1022 results**

→ After Duplicates screening: 659 results

→ After Title screening: 209 results

→ After Abstract screening: 59 results

→ After Full text screening: 38 results

Included studies: 38

**Supplementary Table 1.** This table summarizes the methodological quality and certainty of evidence for each included study, using the GRADE (Grading of Recommendations, Assessment, Development and Evaluations) approach.

| **Study** | **Study Design** | **Risk of Bias** | **Inconsistency** | **Indirectness** | **Imprecision** | **Other Considerations** | **Certainty of Evidence** |
| --- | --- | --- | --- | --- | --- | --- | --- |
| Samanci 2016 | Observational (CSF/serum cytokines, autoantibodies, NSE) | Serious | Not serious | Serious | Serious | None | Very low |
| Samanci 2017 | Observational (CSF/serum cytokines, autoantibodies, NSE) | Serious | Not serious | Serious | Serious | None | Very low |
| ReihamKermani 2008 | Observational (CSF/serum cytokines, autoantibodies, NSE) | Serious | Not serious | Serious | Serious | None | Very low |
| Yetimler 2020 | Observational (CSF/serum cytokines, autoantibodies, NSE) | Serious | Not serious | Serious | Serious | None | Very low |
| Sinclair 2010 | Interventional or hormonal pathway studies | Serious | Not serious | Not serious | Serious | None | Low |
| Skau 2010 | Interventional or hormonal pathway studies | Serious | Not serious | Not serious | Serious | None | Low |
| Westgate 2022 | Interventional or hormonal pathway studies | Serious | Not serious | Not serious | Serious | None | Low |
| Svart 2024 | Neurodegenerative biomarkers (NfL, tau, amyloid) | Serious | Not serious | Not serious | Serious | None | Low |
| Knoche 2023 | Neurodegenerative biomarkers (NfL, tau, amyloid) | Serious | Not serious | Not serious | Serious | None | Low |
| Engel 2023 | Neurodegenerative biomarkers (NfL, tau, amyloid) | Serious | Not serious | Not serious | Serious | None | Low |
| Lecube 2012 | Omics-based proteomic and transcriptomic profiling | Serious | Not serious | Serious | Serious | None | Very low |
| Pandit 2023 | Omics-based proteomic and transcriptomic profiling | Serious | Not serious | Serious | Serious | None | Very low |
| Zanello 2018 | Omics-based proteomic and transcriptomic profiling | Serious | Not serious | Serious | Serious | None | Very low |
| Kranjc 2024 | CGRP and migraine in IIH | Serious | Not serious | Serious | Serious | None | Very low |
| Warner 2007 | Case-control metabolic/endocrine (retinol, androgen axis) | Serious | Not serious | Serious | Serious | None | Very low |
| O'Reilly 2019 | Case-control metabolic/endocrine (retinol, androgen axis) | Serious | Not serious | Serious | Serious | None | Very low |
| Karabork 2024 | CSF inflammation biomarkers case-control | Serious | Not serious | Serious | Not serious | None | Low |
| Edwards 2013 | CSF inflammation biomarkers case-control | Serious | Not serious | Serious | Not serious | None | Low |
| El-Tamawy 2019 | CSF inflammation biomarkers case-control | Serious | Not serious | Serious | Not serious | None | Low |
| Klein 2013 | Endocrine/metabolic observational | Serious | Not serious | Not serious | Serious | None | Low |
| Korsbæk 2024 | Endocrine/metabolic observational | Serious | Not serious | Not serious | Serious | None | Low |
| Dundar 2022 | CSF immune-modulatory markers (Sortilin, IL-33) | Serious | Not serious | Serious | Not serious | None | Low |
| Fahmy 2021 | Inflammation/metabolomics | Serious | Not serious | Not serious | Serious | None | Low |
| Grech 2022 | Inflammation/metabolomics | Serious | Not serious | Not serious | Serious | None | Low |
| Beier 2020 | Prospective observational (biomarker: NfL) | Serious | Not serious | Not serious | Serious | None | Low |
| Ceylan 2021 | Case-control (inflammation markers: NLR, PLR, cytokines) | Serious | Not serious | Serious | Not serious | None | Low |
| Altiokka-Uzun 2015 | Case-control (inflammation markers: NLR, PLR, cytokines) | Serious | Not serious | Serious | Not serious | None | Low |
| Da 2004 | CSF immunology observational | Serious | Not serious | Serious | Not serious | None | Low |
| Altıokka-Uzun 2017 | CSF immunology observational | Serious | Not serious | Serious | Not serious | None | Low |
| Dopper 2016 | CSF hormone and adipokine observational | Serious | Not serious | Serious | Not serious | None | Low |
| Ball 2009 | CSF hormone and adipokine observational | Serious | Not serious | Serious | Not serious | None | Low |
| Abdelghaffar 2022 | CSF hormone and adipokine observational | Serious | Not serious | Serious | Not serious | None | Low |
| Ak 2024 | Case-control (CGRP biomarker study) | Serious | Not serious | Serious | Serious | None | Very low |
| Alimajstorovic et al. 2023 | Case-control + longitudinal metabolomics (CSF and serum) | Serious | Not serious | Not serious | Serious | None | Low |
| Ekizoglu et al. 2012 | Case-control (anti-AQP4 autoantibodies) | Serious | Not serious | Serious | Serious | None | Very low |
| Fahmy et al. 2024 | Case-control (inflammatory biomarkers and MRV) | Serious | Not serious | Not serious | Serious | None | Low |

|  | **FULL TEXT SCREENING** |  |  |
| --- | --- | --- | --- |
| **n** | **Inlcude/Exclude with reason** | **Authors** | **Title** |
| *1* | **included** | *Ekizoglu 2012* | Aquaporin-4 antibodies are not present in patients with idiopathic intracranial hypertension |
| *2* | **included** | *Fahamy 2024* | Association between laboratory markers, clinical and radiological findings in patients with idiopathic intracranial hypertension: case–control study |
| *3* | **included** | *ReihamKermani 2008* | Cerebrospinal fluid concentration of interleukin-6 and interleukin-10 in idiopathic intracranial hypertension |
| *4* | **included** | *Dundar 2022* | Cerebrospinal fluid levels of sortilin-1, lipocalin-2, autotaxin, decorin and interleukin-33 in patients with idiopathic intracranial hypertension |
| *5* | **included** | *Doppler 2016* | Decreased levels of aquaporin-4 in the cerebrospinal fluid of patients with idiopathic intracranial hypertension |
| *6* | **included** | *Ball 2009* | Elevated cerebrospinal fluid (CSF) leptin in idiopathic intracranial hypertension (IIH): evidence for hypothalamic leptin resistance? |
| *7* | **included** | *Engel 2023* | Elevated neurofilament light chain CSF/serum ratio indicates impaired CSF outflow in idiopathic intracranial hypertension |
| *8* | **included** | *Samancl 2017* | Neuron-specific enolase levels as a marker for possible neuronal damage in idiopathic intracranial hypertension |
| *9* | **included** | *Samancl 2016* | Evidence for potential involvement of pro-inflammatory adipokines in the pathogenesis of idiopathic intracranial hypertension |
| *10* | **included** | *Pandit 2024* | Cerebrospinal fluid proteins in idiopathic intracranial hypertension: An exploratory SWATH proteomics analysis |
| *11* | **included** | Edwards 2013 | Increased levels of interleukins 2 and 17 in the cerebrospinal fluid of patients with idiopathic intracranial hypertension |
| *12* | **included** | Zannello 2018 | Inflammatory gene expression signatures in idiopathic intracranial hypertension: possible implications in microgravity-induced ICP elevation |
| *13* | **included** | *Altiokka-Uzun 2015* | Oligoclonal bands and increased cytokine levels in idiopathic intracranial hypertension |
| *14* | **included** | Karabork 2024 | Is increased activator protein 1 in cerebrospinal fluid as a potential biomarker that distinguishes idiopathic intracranial hypertension from multiple sclerosis? |
| *15* | **included** | *Korsbaek 2024* | Metabolic Dysfunction in New-Onset Idiopathic Intracranial Hypertension: Identification of Novel Biomarkers |
| *16* | **included** | *Skau 2010* | Natriuretic pro-peptides in idiopathic intracranial hypertension |
| *17* | **included** | *Beier 2020* | Neurofilament light chain as biomarker in idiopathic intracranial hypertension |
| *18* | **included** | *Svart 2024* | Neurofilament light chain is elevated in patients with newly diagnosed idiopathic intracranial hypertension: A prospective study |
| *19* | **included** | *Knoche 2023* | Neurofilament light chain marks severity of papilledema in idiopathic intracranial hypertension |
| *20* | **included** | *Ceylan 2021* | Neutrophil-to-lymphocyte and platelet-to-lymphocyte ratios as inflammation markers in patients with papilledema due to idiopathic intracranial hypertension |
| *21* | **included** | *Altiokka-Uzun 2017* | Glial and neuronal antibodies in patients with idiopathic intracranial hypertension |
| *22* | **included** | *El-Tamawy 2019* | Oligoclonal bands and levels of interleukin 4, interleukin 10, and tumor necrosis factor alpha in idiopathic intracranial hypertension Egyptian patients |
| *23* | **included** | *Krajnce 2024* | Plasma calcitonin gene-related peptide levels in idiopathic intracranial hypertension: an exploratory study |
| *24* | **included** | *Da 2004* | Polyclonal B-cell expansion in the cerebrospinal fluid of patients with psedotumor cerebri |
| *25* | **included** | *Lecube 2012* | Proteomic Analysis of Cerebrospinal Fluid from Obese Women with Idiopathic Intracranial Hypertension: A New Approach for Identifying New Candidates in the Pathogenesis of Obesity |
| *26* | **included** | *Yetimler 2020* | Serum glial fibrillary acidic protein (GFAP)-antibody in idiopathic intracranial hypertension |
| *27* | **included** | *Westgate 2021* | Systemic and adipocyte transcriptional and metabolic dysregulation in idiopathic intracranial hypertension |
| *28* | **included** | *Ak 2024* | Calcitonin gene-related peptide (CGRP) levels in peripheral blood in patients with idiopathic intracranial hypertension and migrain |
| *29* | **included** | *Warner 2007* | Retinol-binding protein and retinol analysis in cerebrospinal fluid and serum of patients with and without idiopathic intracranial hypertension |
| *30* | **included** | *Fahmy 2021* | Role of tumor necrosis factor-alpha in the pathophysiology of idiopathic intracranial hypertension |
| *31* | **included** | *Dhungana 2009* | Cytokines and Chemokines in Idiopathic Intracranial Hypertension |
| *32* | **included** | *Abdelghaffar 2022* | Sex hormones, CSF and serum leptin in patients with idiopathic intracranial hypertension |
| *33* | **included** | *Alimajstorovic 2023* | Dysregulation of Amino Acid, Lipid, and Acylpyruvate Metabolism in Idiopathic Intracranial Hypertension: A Non-targeted Case Control and Longitudinal Metabolomic Study |
| *34* | **included** | *Klein 2013* | Hyperandrogenism is Associated with Earlier Age of Onset of Idiopathic Intracranial Hypertension in Women |
| *35* | **included** | *O’Reilly 2019* | A unique androgen excess signature in idiopathic intracranial hypertension is linked to cerebrospinal fluid dynamics |
| *36* | **included** | *Sinclair 2010* | Cerebrospinal Fluid Corticosteroid Levels and Cortisol Metabolism in Patients with Idiopathic Intracranial Hypertension: A Link between 11β-HSD1 and Intracranial Pressure Regulation? |
| *37* | **included** | *Westgate 2022* | Increased systemic and adipose 11β-HSD1 activity in idiopathic intracranial hypertension |
| *38* | **included** | *Grech 2022* | Nuclear Magnetic Resonance Spectroscopy Metabolomics in Idiopathic Intracranial Hypertension to Identify Markers of Disease and Headache |
| *39* | *Exlcuded: lack of data* | *Kowarik 2014* | Immune cell subtyping in the cerebrospinal fluid of patients with neurological diseases |
| *40* | *Exlcuded: lack of data* | *Selhorst 2000* | Retinol-binding protein in idiopathic intracranial hypertension (IIH) |
| *41* | *Exlcuded: wrong outcome/s* | *Walker 2017* | A comparison of HMGB1 concentrations between cerebrospinal fluid and blood in patients with neurological disease |
| *42* | *Exlcuded: wrong outcome/s* | *Katz 2002* | Expression of somatostatin receptors 1 and 2 in human choroid plexus and arachnoid granulations - Implications for idiopathic intracranial hypertension |
| *43* | *Exlcuded: wrong outcome/s* | *Kesler 2000* | Idiopathic intracranial hypertension and anticardiolipin antibodies |
| *44* | *Exlcuded: wrong study design* | *Pollak 2015* | The laboratory profile in idiopathic intracranial hypertension |
| *45* | *Exlcuded: wrong study design* | *Samanci 2016* | Trying to Illuminate the Unknown Pathophysiology of Idiopathic Intracranial Hypertension: A Study of Various Biomarkers Including Cytokines and Adipokines |
| *46* | *Exlcuded: wrong study design* | *Luo 2023* | Cerebrospinal fluid glial fibrillary acidic protein-antibody in idiopathic intracranial hypertension: a case report |
| *47* | *Exlcuded: wrong study design* | Yousaf 2022 | Pseudotumor Cerebri Syndrome Without Headache in an Obese Male With Eight Restricted Cerebrospinal Fluid (CSF) Oligoclonal Bands: A Case Report |
| *48* | *Exlcuded: wrong study design* | *Zarei 2023* | Idiopathic intracranial hypertension associated with polycystic ovarian syndrome, sensorineural hearing loss, and elevated inflammatory markers that lead to bilateral blindness: A case report with literature review |
| *49* | *Exlcuded: wrong study design* | *Peng 2007* | Increased ALZ-50 immunoreactivity in CSF of pseudotumor cerebri patients |
| *50* | *Exlcuded: wrong study design* | Inshasi | Intrathecal synthesis of IgG in benign intracranial hypertension: a re-examination |
| *51* | *Exlcuded: wrong study design* | *Yousaf 2022* | Pseudotumor Cerebri Syndrome Without Headache in an Obese Male With Eight Restricted Cerebrospinal Fluid (CSF) Oligoclonal Bands: A Case Report |
| *52* | *Exlcuded: Pediatric population* | *Leker 1998* | Anticardiolipin antibodies are frequently present in patients with idiopathic intracranial hypertension |
| *53* | *Exlcuded: Pediatric population* | *Brettschneider 2011* | Cerebrospinal fluid markers of idiopathic intracranial hypertension: Is the renin-angiotensinogen system involved? |
| *54* | *Exlcuded: Pediatric population* | *Dotan 2013* | Pediatric Pseudotumor Cerebri Associated With Low Serum Levels of Vitamin A |
| *55* | *Exlcuded: Pediatric population* | *Berezovsky 2017* | Cerebrospinal fluid total protein in idiopathic intracranial hypertension |
| *56* | *Exlcuded: Pediatric population* | *Genizi 2023* | High CCL2 Levels Detected in CSF of Patients with Pediatric Pseudotumor Cerebri Syndrome |
| *57* | *Exlcuded: Pediatric population* | Margeta 2015 | Low cerebrospinal fluid protein in prepubertal children with idiopathic intracranial hypertension |
| *58* | *Exlcuded: Pediatric population* | *Dotan 2013* | Pediatric Pseudotumor Cerebri Associated With Low Serum Levels of Vitamin A |
| *59* | *Exlcuded: Pediatric population* | *Tabassi 2005* | Serum and CSF vitamin A concentrations in idiopathic intracranial hypertension |
